# Diagnostic value of platelet‐lymphocyte ratio and hemoglobin‐platelet ratio in patients with rectal cancer

**DOI:** 10.1002/jcla.23153

**Published:** 2020-01-20

**Authors:** Cui‐ju Mo, Zuo‐jian Hu, Shan‐zi Qin, Hua‐ping Chen, Li Huang, Shan Li, Zhao Cao

**Affiliations:** ^1^ Department of Laboratory Medicine First Affiliated Hospital of Guangxi Medical University Nanning China

**Keywords:** diagnostic, hemoglobin‐platelet ratio, platelet‐lymphocyte ratio, rectal cancer

## Abstract

**Background:**

This study aimed to investigate the diagnostic value of platelet‐lymphocyte ratio (PLR) and hemoglobin‐platelet ratio (HPR) combined or not with carcinoembryonic antigen (CEA) in rectal cancer.

**Methods:**

We recruited 235 patients pathologically diagnosed with rectal cancer, 113 patients with benign rectal diseases, and 229 healthy control patients in this retrospective analysis. Then, the correlation between PLR, HPR, and clinicopathological findings was analyzed. Receiver operating characteristic (ROC) curve was used to assess the diagnostic value of PLR and HPR combined or not with CEA in rectal cancer patients.

**Results:**

The levels of PLR, HPR, and CEA were higher in rectal cancer patients than those in the subjects with benign rectal diseases (*P* < .001) and the healthy controls (*P* < .001). Platelet‐lymphocyte ratio and HPR were associated with lymph node metastasis and tumor stage, rather than serosa invasion, distant metastasis, or tumor size. PLR or HPR combined with CEA produced larger area under curve (AUC) (AUC_PLR+CEA_ = 0.75, 95% CI = 0.70‐0.79, AUC_HPR+CEA_ = 0.76, 95% CI = 0.71‐0.80) than PLR (*P* < .0001), HPR (*P* < .0001), or CEA (*P* = .024) alone.

**Conclusion:**

Our results suggest that PLR or HPR combined with CEA can increase diagnostic efficacy and may be a useful diagnostic marker for patients with rectal cancer.

**Abbreviations**
AGR: albumin‐globulin ratio
AUC: area under curve
CEA: carcinoembryonic antigen
CRC: colorectal cancer
HPR: hemoglobin‐platelet ratio
LMR: lymphocyte‐monocyte ratio
NLR: neutrophil‐lymphocyte ratio
PLR: platelet‐lymphocyte ratio
ROC: receiver operating characteristic curve
WBC: white blood cells

## 1 INTRODUCTION

Colorectal cancer (CRC) is a common malignant cancer with high morbidity and mortality worldwide.^1^ About 43 030 new cases of CRC were diagnosed in the USA in 2018.^2^ In China, CRC ranks the third highest morbidity factor and the fifth highest mortality. Most patients with CRC were initially diagnosed with locally advanced tumors, and the prognosis was poor after radical operation.^3^ Early diagnosis can improve the survival rate of patients with CRC. The 5‐year survival rate of early CRC was more than 90.0%, while that of metastatic CRC was only 14.0%.^4^ Rectal cancer accounts for 30.7% of all CRC cases and the incidence of rectal cancer increases with age.^2^, ^5^ Therefore, early screening and diagnosis is an indubitable way to prevent and treat rectal cancer. The identification of a reliable biomarker that can diagnose rectal cancer early is imperative.

Inflammation plays an extremely important role in the process of tumorigenesis and development. Both local and systemic inflammatory reactions can stimulate the immune microenvironment and contribute to the occurrence and development of cancer cells.^6^ Inflammatory response markers include neutrophil, lymphocyte, platelet, albumin, and so on. Platelet can stimulate the growth of tumor cells by aggregating and degranulating in tumor microvessels. Tumor‐related inflammatory mediators can also stimulate platelet elevation.^7^ Lymphocyte is an important component of anti‐tumor immunity. It can distinguish and kill tumor cells or release a series of cytokines to activate anti‐tumor immunity.^8^, ^9^ Being an indicator to reflect the balance between systemic inflammatory response and immune system function that confirmed by several retrospective studies. Platelet‐lymphocyte ratio (PLR) was associated with the diagnosis and prognosis of several malignant tumors, such as gastric cancer,^10^ colorectal cancer,^11^ and pancreatic cancer.^12^ On the other hand, the diagnostic and prognostic role of hemoglobin level has not been clearly defined yet. One study reported that low hemoglobin levels were proposed as parts of a prognostic model regarding cancer‐specific survival in different cancerous diseases.^13^Hematological parameters as indicator to reflect the balance between systemic inflammatory response and immune system function were confirmed by several retrospective studies.

Rectal cancer is a high malignancy with insidious onset and lack of specific symptoms in the early stage. Early screening and diagnosis play an important role in reducing the mortality of rectal cancer. Fecal occult blood test (FOBT) is economical, non‐invasive, and widely used screening important method, while the results are susceptible to the influence of diet and drugs, and it can be detected only when the pathological tissue is bleeding.^14^ Carcinoembryonic antigen (CEA) is a widely used tumor marker, and most malignancies usually have a high concentration of CEA serum. Due to insufficient sensitivity and low organ specificity, CEA cannot be used alone as a cancer screening biomarker. Recent studies have shown that markers of systemic inflammation could be useful biomarkers for the diagnosis of many cancers. As far as we know, there were several retrospective analyses that investigated the relationship between PLR, neutrophil‐lymphocyte ratio (NLR), and albumin‐globulin ratio (AGR) in the prognosis of rectal cancer.^11^, ^15^, ^16^ But rarely have studies assessed the diagnosis role of these hematological parameters in rectal cancer. Therefore, we intend to investigate the role of PLR, hemoglobin‐platelet ratio (HPR), and CEA, which were used alone or in combination, in early screenings and diagnoses of rectal cancer.

## 2 MATERIALS AND METHODS

### 2.1 Patients

Subjects with rectal cancer, benign rectal diseases, and healthy controls were recruited from January 2012 to September 2018, in the First Affiliated Hospital of Guangxi Medical University, China. Two hundred and thirty‐five patients who were newly diagnosed with rectal cancer by histology and then underwent surgical resection were included. All patients were staged according to the seventh edition of the American Joint Committee on Cancer/TNM tumor staging. The exclusion criteria were as follows: (a) treated by radiotherapy and chemotherapy, or did not receive pharmacological treatment; (b) complications with other cancers; (c) had gastroduodenal disease, cardiovascular disease, diabetes mellitus, kidney disease, blood disease, autoimmune disease, or liver disease; and (d) recently received a blood transfusion. One hundred and thirteen patients diagnosed with rectal polyps, rectal adenomas, and rectitis via colonoscopy and histopathology were included in the benign rectal disease group. Two hundred and twenty‐nine healthy individuals were recruited from the physical examination center of the same hospital. No statistical differences were found in gender or age among the three groups. This study was approved by the ethics committee of the First Affiliated Hospital of Guangxi Medical University, China.

### 2.2 Data collection

All data were collected from the hospital's electronic medical records for the first test results of laboratory. Whole blood cell parameters were tested by the Beckmann 780 (Beckman Coulter). The collected data included total number of white blood cells (WBC), platelet values, absolute value of neutrophil, an absolute value of lymphocyte and hemoglobin. The concentration of serum CEA was detected with the Roche E6000 analyzer (Roche Diagnostics). Platelet ‐lymphocyte ratio was calculated as platelet/lymphocyte count. HPR was calculated as hemoglobin/total number of platelets.

### 2.3 Statistical analysis

Normality test was performed using the Kolmogorov‐Smirnov test. Abnormal data were represented as the median with interquartile ranges. A Mann‐Whitney *U* test was used to assess the statistical differences between the two groups, and a chi‐square test was used to analyze the distribution of categorical variables. Data were compared among the three groups by one‐way ANOVA or Kruskal‐Wallis H tests. The specificity, sensitivity, positive predictive value, negative predictive value, area under the curve (AUC), as well as the diagnostic values of CEA, PLR, and HPR in rectal cancer, were estimated with a receiver operating characteristic (ROC) curve. All data were analyzed by SPSS 16.0 (IBM) and MedCalc 15.0 (MedCalc Software). Statistical differences were considered as *P* < .05.

## 3 RESULTS

### 3.1 Clinical characteristics among different groups

**Figure 1:**
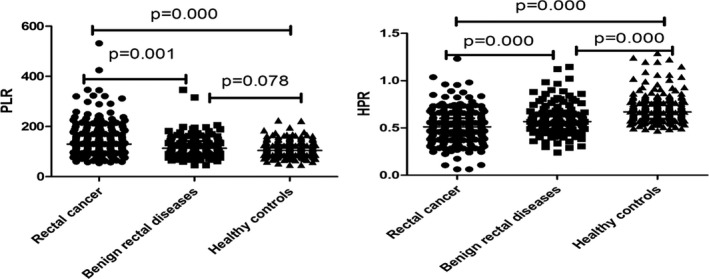
PLR and HPR among three groups

The patient‐related parameters and baseline hematological parameters are shown in Table 1. No significant differences existed in age or gender among the rectal cancer, benign rectal diseases, and healthy control groups. Compared to the benign rectal diseases and healthy control groups, rectal cancer patients had a higher level of WBC, platelet and lower hemoglobin, lymphocyte. The concentration of CEA in the rectal cancer group was significantly higher than that in groups of benign rectal diseases (*P* < .001) and healthy control (*P* < .001). As shown in Table 1 and Figure 1, the median of PLR in the rectal cancer group was significantly higher than that in the benign rectal diseases(*P* < .001) or healthy control (*P* < .001) groups, while there were no statistical differences in PLR between the benign rectal diseases and healthy control groups (*P* = .078). The median HPR levels in the two disease group were lower than that in the healthy control group (rectal cancer vs benign rectal diseases, *P* < .001; rectal cancer vs healthy controls, *P* < .001; benign rectal diseases vs healthy controls, *P* < .001).

**Table 1:** Clinical characteristics among rectal cancer, benign rectal diseases, and healthy control groups

	Rectal cancer	Benign rectal diseases	Healthy controls	a *P‐*value	b *P‐*value	c *P‐*value
Number	235	113	229			
Gender (Male, %)	140 (59.6%)	56 (49.6%)	133 (58.1%)	.078	.743	.136
Age (y)	55.3 ± 12.3	52.7 ± 12.2	54.6 ± 9.5	.042	.523	.132
WBC (×10^9^/L)	6.45 (5.59, 7.67)	6.00 (5.21, 7.22)	6.04 (5.09, 6.73)	.020	.000	.208
Hemoglobin (g/L)	126.00 (113.90, 136.10)	128.50 (118.90, 136.40)	147.30 (139.90, 154.50)	.136	.000	.000
Platelet (×10^9^/L)	250.80 (214.90, 294.00)	224.40 (185.30, 253.50)	222.40 (194.50, 242.90)	.000	.000	.824
Lymphocyte (×10^9^/L)	1.89 (1.53, 2.34)	1.92 (1.58, 2.29)	2.10 (1.76, 2.42)	.723	.004	.020
HPR	0.51 (0.42, 0.60)	0.57 (0.49, 0.68)	0.67 (0.60, 0.77)	.000	.000	.000
PLR	129.56 (96.61, 171.60)	113.41 (89.00, 141.64)	104.10 (88.33, 126.15)	.001	.000	.078
CEA (ng/mL)	2.83 (1.59, 4.58)	1.65 (1.19, 2.38)	1.15 (0.65, 2.02)	.000	.000	.000

Abbreviations: CEA, carcinoembryonic antigen; HPR, hemoglobin‐platelet ratio; PLR, platelet‐lymphocyte ratio; WBC, white blood cell.

a *P* < .05; rectal cancer group vs benign rectal diseases group (Mann‐Whitney nonparametric *U* test).

b *P* < .05; rectal cancer group vs healthy controls (Mann‐Whitney nonparametric *U* test).

c *P* < .05; benign rectal diseases group vs healthy controls (Mann‐Whitney nonparametric *U* test).

### 3.2 Correlation between PLR, HPR, CEA, and clinicopathological features in rectal cancer

As shown in Table 2, the median of HPR in male patients was higher than that in female patients (*P* < .001), while there were no statistical differences in PLR, CEA between male and female patients (*P* = .770 for PLR; *P* = .506 for CEA). PLR and HPR were both related to lymph node metastasis and tumor stage; however, they were not associated with serosa invasion, distant metastasis, or tumor size. A significant difference of CEA level was observed according to the classification of serosa invasion and tumor size; however, there were no differences of CEA concentration in the category of lymph node metastasis, distant metastasis, and stage.

**Table 2:** Correlation between clinicopathological features and PLR, HPR, CEA in rectal cancer

	N	PLR	*P*	HPR	*P*	CEA	*P*
Gender							
Male	140	130.89 (96.00, 171.23)	.770	0.55 (0.44, 0.64)	.000	2.86 (1.68, 4.53)	.506
Female	95	128.16 (103.75, 175.37)		0.47 (0.38, 0.55)		2.74 (1.45, 4.83)	
Tumor invasion (T stage)							
T1 + T2	101	121.29 (93.18, 160.96)	.064	0.52 (0.44, 0.61)	.055	2.66 (1.68, 3.90)	.047
T3 + T4	134	134.80 (98.82,185.33)		0.48 (0.38, 0.59)		3.06 (1.57, 5.91)	
Lymph node metastasis (N stage)							
N0	146	125.16 (96.13, 160.30)	.036	0.52 (0.44, 0.63)	.010	2.74 (1.57, 4.25)	0.359
N1‐N3	89	141.78 (98.24, 196.68)		0.47 (0.37, 0.57)		2.95 (1.64, 5.60)	
Distant metastasis (M stage)							
M0	231	129.56 (96.60, 171.23)		0.51 (0.42, 0.60)		2.79 (1.59, 4.56)	
M1	4	167.18 (92.50, 286.05)		0.43 (0.29, 0.69)		5.14 (1.75, 10.53)	
Tumor size (cm)							
<5	128	127.80 (96.34, 161.69)	.398	0.51 (0.43, 0.61)	.108	2.62 (1.54, 3.95)	.021
≥5	107	130.83 (98.35, 195.03)		0.50 (0.38, 0.58)		3.18 (1.67, 6.52)	
Stage							
I + II	161	123.87 (94.50, 160.96)	.005	0.53 (0.44, 0.61)	.000	2.74 (1.60, 4.26)	.317
III + IV	74	147.30 (108.62, 209.56)		0.45 (0.37, 0.57)		3.07 (1.57, 5.66)	

### 3.3 Diagnostic efficacy of PLR, HPR, and CEA, alone or in combination to differentiate rectal cancer from benign rectal diseases

**Figure 2:**
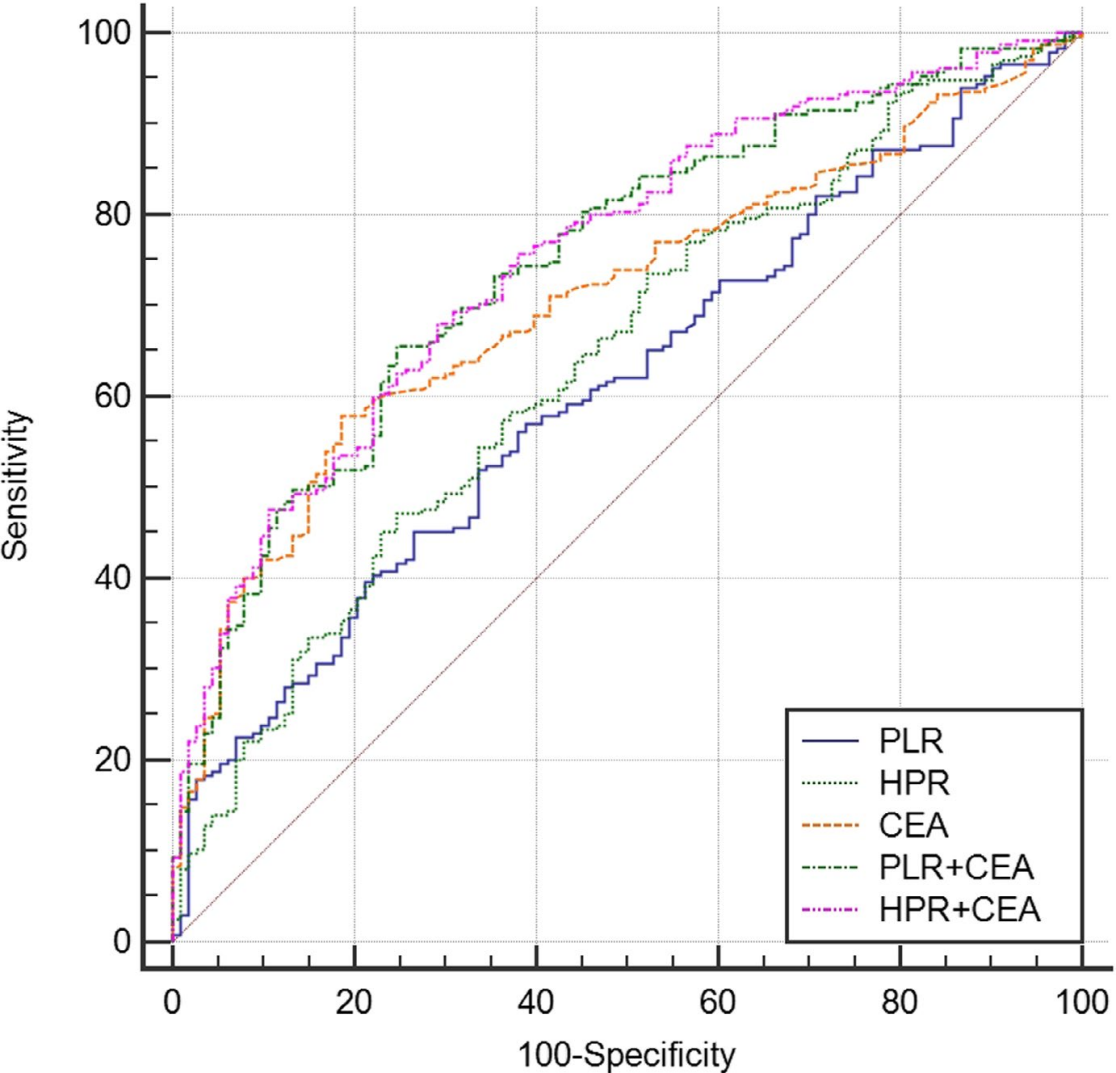
Diagnostic value of NLR, PLR, and CEA used alone or in combination, for distinguishing rectal cancer from benign rectal diseases

The diagnostic accuracy of PLR, HPR, and CEA for the prediction of histologic severity is shown in Table 3 and Figure 2. The sensitivities of PLR, HPR, and CEA were 45.1%, 47.23%, and 57.87%, respectively, while the AUC was 0.61, 0.64, and 0.70, respectively. The sensitivity was increased in the combination of PLR or HPR and CEA (65.53% for PLR + CEA; 68.09% for HPR + CEA). Similarly, the AUC value of combination for PLR and CEA (0.75, 95% CI = 0.70‐0.79) was larger compared to PLR (*P* < .001) or CEA (*P* = .024) alone. The combined use of HPR and CEA resulted in greater AUC (0.76, 95% CI = 0.71‐0.80) than using HPR (*P* < .001) or CEA (*P* = .026) alone.

**Table 3:** Diagnostic efficiency of PLR, HPR, and CEA used alone or in combination to differentiate rectal cancer from benign rectal diseases

	Cut‐off value	Sensitivity	Specificity	+LR	−LR	AUC
PLR	135.11	45.11	73.45	1.70	0.75	0.61 (0.56‐0.66)
HPR	0.49	47.23	75.22	1.91	0.70	0.64 (0.59‐0.69)
CEA	2.52	57.87	81.42	3.11	0.52	0.70 (0.66‐0.75)
PLR + CEA	0.34	65.53	75.22	2.64	0.46	0.75 (0.70‐0.79)
HPR + CEA	0.35	68.09	70.8	2.33	0.45	0.76 (0.71‐0.80)

Abbreviations: +LR, positive likelihood ratio; −LR, negative likelihood ratio.

## 4 DISCUSSION

Chronic inflammation contributes to carcinogenesis and increases cancer risk, including CRC.^17^ Colorectal cancer is a commonly diagnosed cancer. The International Agency for Research on Cancer pointed out that CRC places tremendous economic pressure on society and families. It is of great significance to seek screening and diagnosis measures. Currently, the main methods used to detect and diagnose CRC are colonoscopy and biopsy, though these are not routine in physical examinations and are not suitable for early screening. Research on the role of blood routine parameters used for the early diagnosis and prognosis of malignant tumors is increasing. The most studied elements are platelet,^18^ lymphocyte,^19^ neutrophil,^20^ and related parameters; PLR, NLR, and lymphocyte‐monocyte ratio (LMR) are widely used in the early diagnosis and prognosis of cancer.

In our current retrospective analysis, we used blood routines and their related parameters PLR and HPR combined or not with CEA to diagnose rectal cancer. The results showed that PLR in the rectal cancer group was significantly higher than that in the benign rectal diseases and healthy control groups, which was in agreement with previous findings.^11^, ^21^, ^22^, ^23^, ^24^ Peng et al found that PLR levels in CRC cases were remarkably higher compared to healthy controls.^21^ A study by Emir also reported that patients with CRC had significantly higher PLR values than patients with colorectal polyps and healthy controls.^22^ Platelet‐lymphocyte ratio can also be used as a prognostic marker in CRC, with high PLR associated with decreased overall survival and progression‐free survival.^11^, ^23^ Jia et al reported that PLR levels were higher in CRC patients and also indicated that PLR was predominantly related to the different stages of CRC development.^24^ Our study also found that PLR was related to lymph node metastasis and tumor stage, coinciding with previous research conclusions.

Research has proven that anemia and thrombocytosis may occur in cancer patients. Growing tumors induce thrombocytosis by secretion of inflammatory cytokines, which may also cause bone marrow suppression and disorders of iron metabolism, resulting in tumor‐induced anemia.^25^, ^26^ Low hemoglobin levels contribute to tumor hypoxia which is responsible for enhanced tumor growth; in addition, anemia can promote angiogenesis and genomic mutations in cells.^27^ Serta et al^28^ observed the level of hemoglobin was lower in patients with CRC compared to a control group. Several studies have demonstrated that preoperative hemoglobin levels were related to the prognosis of tumors, patients with pretreatment hemoglobin 12 g/dL, or less potentially yielding worse outcomes in breast cancer,^29^ ovarian cancer,^30^ and transitional cell carcinoma.^31^ Consistent with previous studies, our research suggested that lower hemoglobin concentrations were found in rectal cancer patients, while there was no statistical difference between the rectal cancer and benign rectal diseases groups. But, compared to the benign rectal diseases and healthy control groups, the value of HPR in the rectal cancer group was lower. A previous study used HPR to prognosticate the oncological outcomes of bladder cancer, pointing out that low HPR and low hemoglobin correlated with poor overall survival and worse cancer‐specific survival.^32^ Our current finding is the first study using the HPR value to distinguish rectal cancer from benign rectal diseases and healthy controls. Furthermore, our finding shows that the median of HPR in male patients was higher than that in female patients, and it may be related to low hemoglobin concentrations in female patients.

Carcinoembryonic antigen is a broad‐spectrum tumor marker commonly used in the diagnosis of gastrointestinal cancer. Here, our results showed that a higher concentration of CEA was found in rectal cancer than in benign rectal diseases and healthy controls. In an ROC curve analysis in comparison to benign rectal diseases, the diagnostic value of PLR or HPR combined with CEA produced larger AUC than using PLR, HPR or, CEA alone. Wu et al reported that PLR combined with CEA can produce larger AUC in gastric cancer diagnosis.^10^ For early‐stage CRC, Peng et al found that PLR combined with CEA provided a higher diagnostic efficacy than PLR or CEA alone and could be used as a CRC diagnostic biomarkery.^21^ According to the results of previous studies and our findings, combining PLR or HPR with CEA may be a promising early diagnosis biomarker for rectal cancer.

Although previous research focused on the prognostic role of hematological parameters in rectal cancer, few studies evaluated their early diagnostic value. Our findings demonstrated the diagnostic roles of PLR and HPR in rectal cancer patients treated with surgery. However, several limitations should also be concerned. First, our study is a retrospective analysis, and selection bias cannot be ruled out completely. Second, we only focused on preoperative hematologic markers; other factors, such as eating habits, smoking, and genetics, were not taken into account, which may affect the final results. Third, all subjects were Asians from a single hospital, and some variations in the rations might be dependent of race. Finally, patients treated by radiotherapy and chemotherapy were excluded, while patients treated with steroids were not excluded which might affect the results. Therefore, prospective study design, larger sample size, and multi‐center clinical study are demanded in future.

In conclusion, we found that PLR and HPR were significantly associated with rectal cancer and its lymph node metastasis, tumor stage. The combination of PLR or HPR with CEA can increase diagnostic efficacy and may be a useful diagnostic marker for distinguishing rectal cancer from benign rectal diseases.
